# A Chinese Version of the Caring Dimensions Inventory: Reliability and Validity Assessment

**DOI:** 10.3390/ijerph18136834

**Published:** 2021-06-25

**Authors:** Lai-Kun Tong, Ming-Xia Zhu, Si-Chen Wang, Pak-Leng Cheong, Iat-Kio Van

**Affiliations:** 1 Research Management and Development Department, Kiang Wu Nursing College of Macau, Macau 999078, China; chingco@kwnc.edu.mo; 2 Education Department, Kiang Wu Nursing College of Macau, Macau 999078, China; zmx@kwnc.edu.mo (M.-X.Z.); sichen@kwnc.edu.mo (S.-C.W.); joecheong@kwnc.edu.mo (P.-L.C.)

**Keywords:** caring dimensions inventory, reliability, validity, nurse, China

## Abstract

Caring is central to nursing practice. Chinese nurses take care for the health of about 20% of the world’s population and their perception of caring is critical. However, until recently, instrument specifically designed to measure the caring of nurses in China was not found. Caring dimensions inventory (CDI) is one of the most frequently used instrument when measuring caring and is applicable to nurses from different cultures. The aim of this study is to test the validity and reliability of the Chinese version of the 25-item CDI. The English version of CDI was translated into Chinese according to the guidelines of the World Health Organization. Content validity was conducted among seven senior nurses from different institutions in different cities in China. A convenience sample of nurses from 11 cities in China was employed. Exploratory factor analysis and confirmatory factor analysis of the CDI was carried out using a sample of 880 nurses. The overall content validity index was 0.98. Three factors (Nurturance, Collaboration, Skill) were identified in exploratory factor analysis and were confirmed by confirmatory factor analysis. The three-factor solution explains 70.15% of the total variance. The Cronbach alpha for overall the CDI was 0.97. This study demonstrated that the Chinese version of the CDI showed satisfactory reliability and validity, indicating that it could be a useful measurement to assess nurses’ perception of caring in China.

## 1. Introduction

International nursing scholars have asserted that caring is the essence of nursing [1,2,3,4]. Scholars in different historical periods and cultural backgrounds have different understandings of the concept of caring in nursing [5]. Watson emphasized that caring is a kind of spiritual experience of interpersonal interaction in a specific time and situation, and the establishment of a good nurse–patient interaction can promote the patient’s physical recovery [2]. Leininger defined caring as a nurse who provides assistance, support, and facility to the needs of patients and their families based on their unique cultural backgrounds [3]. Nurses can make use of different theoretical models to carry out caring according to different cultures and situations. The most commonly used theoretical models are Watson’s Caring Model and Roach’s 5C’s of Caring Theory. Watson’s 10 carative factors are referred to as the interventions of the model [2]. Roger proposed that nurses need to have compassion, confidence, competence, commitment, and conscience to perform caring [6].

Caring is central to nursing practice. An umbrella review shows that both nurses and patients have expectations about caring relationships [7]. Caring not only improve patients satisfaction [8], but also mental well-being of patients and nurses [9], as well as physical well-being of patients [10]. The understanding of caring will essentially influence the way nurses provide care, and the quality of care delivered; more importantly, how nurses perceive care affects how they understand the concept of nursing [11]. Caring is an important concept in nursing, however, it is an elusive concept and difficult to assess [12]. Even though any measurement of caring is only “an indicator of something deeper”, it may allow researchers to be more explicit [13].

Academic researchers have developed a number of tools for measuring caring [14,15]. Most of these instruments are designed based on sound theoretical frameworks such as Mayeroff’s eight caring ingredients (e.g., the Caring Ability Inventory [CAI] [16]), Watson’s theory of human caring (e.g., Caring Behavior Inventory [CBI] [17]), or a combination of different theories (e.g., Caring Attributes, Professional Self, and Technological Influence Instrument [18], the Caring Dimension Inventory [CDI] [19]).

Chinese nurses take care for the health of about 20% of the world’s population [20], and their perception of caring is critical. Therefore, Chinese nurses’ perception of caring needs to be assessed by an appropriate Chinese language tool. However, until recently, we have not found any instrument specifically designed to measure the caring of nurses in China. To fill the gap, we decided to translate and modify existing instruments.

CAI can be applied to anyone, so it does not well reflect a nurse’s caring for patient in clinical practice. CBI is a self and observer rating scale to measure caring behavior, so it is mostly overt behavior and attitude, covert behavior and attitude are not included. CDI is one of the most frequently used instrument when measuring caring [14,15], it has a small number of items [14] and is applicable to nurses from different cultures [21,22,23]. CDI would appear to be an appropriate caring inventory to use within the Chinese context. Therefore, we choose as a potential tool to measure Chinese nurses’ perception of caring.

CDI is a 25-item, 5-point Likert scale designed to measure nurses’ perception of caring [19]. Items on the scale are rated from 1 (strongly disagree) to 5 (strongly agree). Scoring is accomplished by summing scores for items. The total score ranges from 25–125. For the English version, the Cronbach’s alpha was 0.91 for the total scale [19].The purpose of the study was to test the validity and reliability of the Chinese version of CDI.

## 2. Materials and Methods

### 2.1. Ethic

The Research Management and Development Department of Kiang Wu Nursing College of Macau provided ethical approval (reference no: 2019APR01). The inventory had no copyright and the developers of the instrument provided their authorization to use it.

### 2.2. Participants

A convenience sample of nurses from 11 cities in China, including Guangzhou, Shenzhen, Zhuhai, Foshan, Dongguan, Zhongshan, Jiangmen, Huizhou, Zhaoqing, Hong Kong, and Macau was surveyed. The nurses recruited for this study were as follows: who were working in hospitals, clinics, schools, elderly service institutions or nursing homes in the above 11 cities, who had passed the probationary period, who were able to read and write Chinese, and who were willing to participate in the study.

### 2.3. Procedures

We translated the CDI into Chinese according to the guidelines of the World Health Organization [24]. We followed a standard procedure in five stages: (1) Forward translation: translation was performed by a native Chinese speaker who has studied and lived in the UK for many years. (2) Expert panel: The draft Chinese translation of the CDI was reviewed by an expert committee comprised of the expert in caring, the experts in nurse, the expert in public health, the expert in translation, and the translator. Experts’ opinions were collected via email and then discussed face to face. The research team put together all the suggestions and revised the draft. A complete translated version of the scale was formed after repeated consultation with experts. (3) Back-translation: A postdoctoral fellow from the UK, who had no knowledge of the scale, carried out a back-translation of the Chinese version into English. The above expert panel steps were carried out for the back-translation version of the scale until a satisfactory version was completed. (4) Pre-testing and cognitive interviewing: Content validity was conducted among seven senior nurses from different institutions in different cities. The content validity index (CVI) range of each item in the scale is 0.86–1.00. The average scale CVI was 0.98, which is above the acceptable value. The expression of some items was improved in the scale according to expert advice. The pre-final version was distributed online to one nurse in each of the 11 cities. The research team interviewed each nurse via social software (WeChat) to collect feedback on the intelligibility, usability, applicability, and completeness of the scale. According to the data and feedback, the scale was revised into the final version. (5) Test of the final version: Further testing of the adapted version was conducted in 11 cities through an online questionnaire.

### 2.4. Data Collection

The research team contacted a questionnaire collector in each city, who was responsible for collecting the questionnaire in the city. The questionnaire collector works in a hospital, university or nursing professional group. The research team produced electronic poster for the study. The questionnaire collectors distributed poster to relevant institutions in their cities or social platforms commonly used by nurses. The poster contains a link to the online questionnaire and a QR code. The online questionnaire included a cover letter that addressed the purpose and importance of the study. After reading the instruction letter, the participants clicked the “Agree to participate” option before filling in the questionnaire. The data were mainly collected from 19 May to 7 August 2020.

### 2.5. Data Analysis

We planned to perform confirmatory factor analysis (CFA) on the scale. CFA requires a minimum of 50 samples [25], preferably 800 [26]. We planned to collect 80 questionnaires in each city, but the number of questionnaires collected online is difficult to control, so after the data collection was completed, SPSS 26.0 was used to randomly select 80 samples for analysis in cities that had collected more than 80 samples.

The CVI was used to determine the content validity [27]. Items with a CVI of 0.80 or higher could be considered evidence of good content validity. SPSS 26.0 was used for internal consistency analysis and exploratory factor analysis (EFA). The Cronbach’s alpha was used to assess the internal consistency reliability. The Cronbach’s alpha of 0.80 and above is good [28]. The mean and standard deviations of item were used to provide information about item difficulty for judgment and endorsement level [29]. Construct validity was assessed by EFA using principal components analysis (PCA) with Varimax rotation. Prior to the test, the Kaiser-Meyer-Olkin (KMO) and Bartlett’s test of spherical (BTS) were used to determine whether the sample data were suitable for EFA. KMO is greater than 0.6 and BTS must be significant at α < 0.05, indicating that the data were sufficient to proceed for EFA [28]. The number of factors to be extracted was guided by Kaiser’s criterion (Eigenvalue ≥ 1), the scree plot test (above the break), the number of items (>3) [30]. Items with factor loadings < 0.50 were deleted. If items with loadings were greater than 0.50 on more than one factor, the item was placed with the factor that it is most closely related to conceptually [31]. The Cronbach’s Alpha for each factor of 0.70 and above is good [32].

We then performed CFA with maximum likelihood factoring to confirm the exploratory model. Another group of samples were randomly selected for CFA analysis using the same method of EFA. CFA was performed using AMOS 22.0. The following goodness-of-fit indices were used to assess the model: Normed fit index (NFI), Incremental Fit Index (IFI), Tucker–Lewis index (TLI), Comparative Fit Index (CFI), and Root Mean Square Error of Approximation (RMSEA) [33]. Model fit is acceptable if NFI ≥ 0.90, IFI ≥ 0.90, TLI ≥ 0.90, CFI ≥ 0.90, and RMSEA ≤ 0.10 [34].

## 3. Results

### 3.1. Characteristics of Participants

Demographic data of nurses are shown in Table 1. Ninety-five percent of subjects were female and more than half (64.4%) were married. The mean age was 34.3 (S.D. = 9.7) with a range from 18 to 69 years old. About half of the subjects had more than 10 years of job experience (48.2%) and nearly three-fifths had a bachelor’s degree (59.5%). The sample mean score of CDI was 107.35 (S.D. = 14.79). As shown in Table 1, males had higher scores, above 40 years old rated higher than other age groups, married nurses scored higher than single nurses.

### 3.2. Validity

#### 3.2.1. Content Validity

The CVI was used to determine the content validity. Table 2 presents the item CVI ranging from 0.86 to 1.00 and the total CVI was 0.98. Item 2, item 4, and item 16 had the lowest CVI of 0.86. For item 2, one expert believed that making a nursing record about a patient was not relevant to the practice of humanistic caring. For item 4, one expert believed that “as a person” could easily produce ambiguity in Chinese context. For item 16, one expert pointed out that sharing personal problems with a patient is not in line with Chinese culture. After discussion and consensus among panel members and researchers, item 2 and 16 were retained in the Chinese version, and item 4 was modified as recommended by the experts.

#### 3.2.2. Construct Validity

The EFA was performed to test the factor structures of CDI. The KMO value was 0.97 and BTS significant with a *p*-value of <0.001, indicating that principal component analysis was appropriate. We performed the principal component analysis with Varimax rotation. A three-factor solution explaining 70.15% of the total variance was obtained, but item 5, 15, 21, and item 24 cross-loaded on two factors. placing the item 24 with the Factor 1, item 15 and item 21 with the Factor 2, item 5 with the Factor 3 that it is most closely related to conceptually. The percentages explained by each factor were 31.36% (Nurturance), 20.27% (Collaboration), and 18.52% (Skill) respectively. The item loadings are presented in Table 3.

#### 3.2.3. Confirmatory Factor Analysis

**Figure 1:**
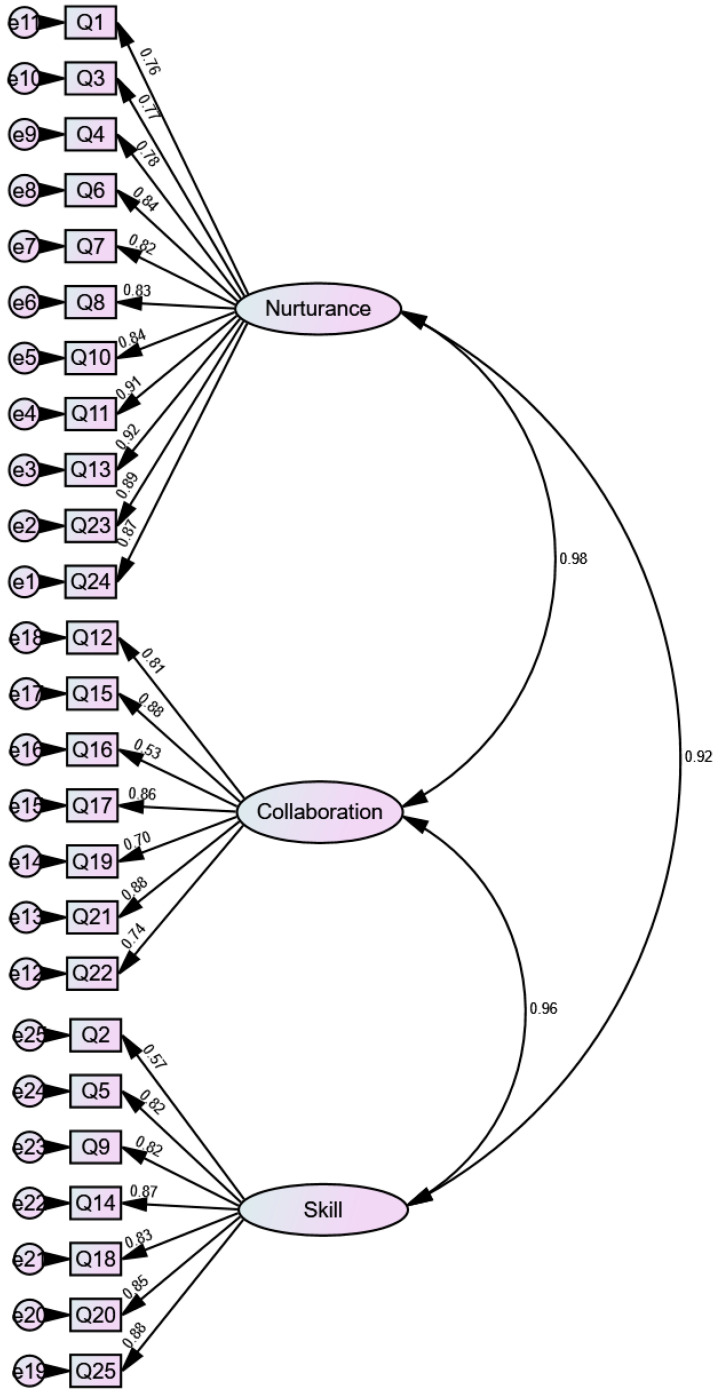
CFA for the CDI with standardized loadings.

Estimation of model fit of CFA using 3-factor model was based on maximum likelihood method. The results of the CFA revealed good model fit of the CDI: NFI was 0.90, IFI was 0.91, TLI was 0.90, CFI was 0.91, and RMSEA was 0.09. Standardized factor loadings are displayed in Figure 1.

### 3.3. Reliability

The Cronbach’s alpha was used to assess the internal consistency reliability. The Cronbach’s alpha for overall CDI was 0.97. All three factors on the CDI had a high rating for reliability (Table 4). The means and standard deviation of each item are presented in Table 2. Item 23 was well endorsed by the nurses in the study with mean score of 4.52, while item 2 was least endorsed with mean score of 3.83.

## 4. Discussion

CDI has proven to be a valid and reliable instrument for assessing nurses’ perception of caring [21,22,23]. But until now, the translation of the Chinese version has never been validated. The purpose of this study was to translate the CDI and test its psychometric properties (reliability and validity) in Chinese nurses. This study demonstrated that the Chinese version of the CDI showed satisfactory reliability and validity, indicating that it could be a useful measurement to assess nurses’ perception of caring in China.

The CVI for the Chinese version of the CDI was high, indicating good content validity. The rates agreed that all items were clear, applicable, and relevant to assess the perception of caring. Item 4 showed the lowest CVI. In English-speaking countries, “as a person” is a common and easily understood expression, but not in China. Experts believed that “as a person” could easily produce ambiguity in Chinese context, so they suggested that the patient should be regarded as a complete person, normal person, or an independent individual. Item 4 of the Chinese version was modified as recommended by the experts.

In this study, the Chinese version of CDI demonstrated high internal consistency (Cronbach’s alpha = 0.97), which is higher than the original English version (Cronbach’s alpha = 0.91) [19] and the Persian version (Cronbach’s alpha = 0.86) [22].

The aspect of nursing work in China which was considered to be most caring was item 23 (providing privacy for a patient), while in the UK and Spain it was item 13 (listening to a patient) [19,21]. This may be due to the fact that this study was conducted later than that of the UK and Spain, and the increasing emphasis on patient privacy [35]. Item 2 (making a nursing record about a patient) was considered the least caring aspect of nursing work in China, compared with item 16 (sharing your personal problems with a patient) in the UK [19] and item 1 (assisting a patient with an activity of living) in Jordan [36]. The difference in the ranking of the items indicates that nurses from different countries ascribe a different level of importance to caring. The reasons for the differences may be cultural, social value, and institutional differences between countries [37]. This is because nurses in China believe that caring is reflected in qualified professional knowledge, attitude, and skills, and provide support for patients [38], while writing medical records is just a daily job, which has nothing to do with caring. What is interesting is that item 16 was the least related dimension of caring in different countries, such as the UK, Turkey, and Saudi Arabia [19,37,39]. In fact, Chinese nurses scored relatively low on item 16, only 0.11 higher than item 2. Like nurses in other parts of Asia, Chinese nurses are less likely to express their feelings in public [40]. At first one expert concerned that item 16 might not be applicable to Chinese culture, but it was tested that it could be retained in the final version.

There are often differences in the factor structure of the scale when it is tested under different cultural backgrounds [41,42]. To better establish a factor solution for Chinese nurses, we proposed a modified factor structure according to the results of EFA. According to the results of EFA in this study, three factors were obtained, and labeled Nurturance, Collaboration, Skill. (1) Nurturance means that nurses care for patients in a manner that respects the uniqueness and value of each individual [43]. Caring is “the moral ideal of nursing whereby the end is protection, enhancement, and preservation of human dignity” [44]. Respectful interpersonal relationships are essential to preserving human dignity [45]. Nurses’ ability to offer patients unconditionally acceptance is an antecedent for Nurturance [43]. Ryan proposes that nurses should take care of patients “in their world, not mine” [46]. (2) Collaboration signifies the collaborative work of patients, nurses, and other multidisciplinary team members to promote high-quality patient outcomes [47]. Transpersonal caring is one of the three main elements of Watson’s theory [48]. The term transpersonal is defined as “an intersubjective human-to-human relationship” [48]. Watson pointed out that nurses need to provide patients with ”supportive, protective, and (or) physical, societal, and spiritual environment” [48]. This requires nurses to cooperate in performing caring. The participation of patients, family members, and health professionals in a cooperative and coordinated manner is the guarantee of high quality and safe health care [49]. Nurses are involved in collaborations ranging from assisting patients with activities of daily living to individual, group or family therapy [50]. (3) Skill indicates basic clinical nursing skills. Although technical knowledge and skills are not integral features of nursing, technical competence is an important aspect of nursing [23]. High quality nursing is a combination of technical competence and psychological care [51].

The number of factors in the Chinese version is different from that in the original English version [52]. The researches claimed that four factor model and five factor model were found to fit acceptably well in the original English version [52]. However, some factors with fewer than three items were extracted in the original English version [52], indicating that these factors are weak and unstable [53]. Although the English version did not mention the Cronbach’s alpha of these unstable factors, the analysis in the Persian version showed that two items were loaded on factor 5 with a relatively low Cronbach’s alpha of 0.31 [22]. The results of this study suggest that the three-factor model may be more appropriate. First, the results of the EFA show that there are no cross-loading above 0.5 and each factor has at least five variables with high loadings (>0.5). Second, the results of the CFA demonstrated satisfactory goodness-of-fit between the data and the factor structure with high item loadings. Third, all three factors on the CDI had a high rating for reliability.

The strength of this study is that it adopts a multistep translation method supported by existing evidence rather than a simple translation/back-translation process [24,54]. However, some limitations must be considered interpreting these findings. First, in this study, one translator was responsible for forward translation and back-translation, respectively. Although the research team organized an expert panel and a team of content validity experts to check the quality of scale translation, the translation scale might not reach the optimal quality. Second, the proportion of male nurses in our sample was less than 5%, so the results may not be generalized to male nurses. Future work should further validate the Chinese version of the CDI with a more representative and larger sample. Third, the stability and reliability of CDI over time cannot be determined without a test-retest reliability analysis. The test-retest reliability of the Chinese version of the CDI should be examined in the future. Forth, only factor analysis was used to test the CDI construct validity. Construct validity alone is not enough to determine the validity of the scale, further validity tests, such as criterion validity, are needed.

## 5. Conclusions

The CDI was successfully translated and culturally adapted into Chinese. The Chinese version of the CDI showed satisfactory reliability and validity among Chinese nurses. Results also reveal three factors underlying nurses’ perception of caring—Nurturance, Collaboration, Skill.

## Figures and Tables

**Table 1:** Summary of demographic data, and CDI mean score (N = 880).

Variable		N (%)	CDI Score	
			Mean	S.D.
Overall		880 (100)	107.35	14.79
Gender				
	Female	837 (95.1)	107.61	14.64
	Male	43 (4.9)	102.21	16.89
Age (years) Range: 18–69, Mean = 34.3 (S.D. = 9.7)				
	≤25	164 (18.6)	108.10	12.64
	26–30	230 (26.1)	106.63	16.03
	31–40	276 (31.4)	106.61	17.05
	≥41	210 (23.9)	108.51	11.33
Education				
	College degree or bellow	295 (33.5)	107.40	13.92
	Bachelor	524 (59.5)	107.26	15.35
	Master or above	61 (6.9)	107.89	14.23
Marital status				
	single	291 (33.1)	107.33	13.01
	married	565 (64.2)	107.40	15.35
	other	24 (2.7)	106.29	21.15
Job experience (years) Range: 0–44, Mean = 12.8 (S.D. = 9.6)				
	<1	6 (0.7)	104.50	16.33
	1–3 years	129(14.7)	107.13	12.66
	3.1–6 years	155 (17.6)	107.60	15.67
	6.1–10 years	166 (18.9)	106.85	15.66
	>10	424 (48.2)	107.56	14.75

**Table 2:** The content, content validity index (CVI), means, standard deviations for CDI items (N = 880).

Item	Item Content		CVI	Mean	S.D.
	English Version	Final Chinese Version			
Q1	Assisting a patient with an activity of living	協助病人進行日常活動	1.00	4.31	0.76
Q2	Making a nursing record about a patient	書寫病人的護理記錄	0.86	3.83	1.06
Q3	Feeling sorry for a patient	對病人的痛苦感同身受	1.00	4.32	0.79
Q4	Getting to know the patient as a person	視病人為一個完整的人	0.86	4.45	0.74
Q5	Explaining a clinical procedure	解釋臨床程序	1.00	4.26	0.79
Q6	Being neatly dressed when working with a patient	接觸病人時保持衣著整齊	1.00	4.37	0.72
Q7	Sitting with a patient	與病人同坐(平等相處)	1.00	4.32	0.76
Q8	Exploring a patient’s lifestyle	深入瞭解病人的生活方式	1.00	4.30	0.76
Q9	Reporting a patient’s condition to a senior nurse	向資深護士報告病人的情况	1.00	4.17	0.81
Q10	Being with a patient during a clinical procedure	進行臨床程序期間陪伴病人	1.00	4.30	0.71
Q11	Being honest with a patient	真誠對待病人	1.00	4.51	0.64
Q12	Organizing the work of others for a patient	組織其他人員(所有人)為病人服務	1.00	4.26	0.76
Q13	Listening to a patient	聆聽病人	1.00	4.51	0.64
Q14	Consulting with the doctor about a patient	向醫生諮詢有關病人的情况	1.00	4.29	0.75
Q15	Instructing a patient about an aspect of self-care	指導病人自我照顧	1.00	4.40	0.67
Q16	Sharing your personal problems with a patient	與病人分享個人困擾	0.86	3.94	1.12
Q17	Keeping relatives informed about a patient	讓病人家屬持續瞭解病情	1.00	4.31	0.71
Q18	Measuring the vital signs of a patient	測量病人的生命體徵	1.00	4.27	0.80
Q19	Putting the needs of a patient before your own	病人需要先於自己	1.00	4.08	0.90
Q20	Being technically competent with a clinical procedure	勝任臨床程序的技術要求	1.00	4.29	0.76
Q21	Involving a patient in his or her care	讓病人參與到自己的健康照顧當中	1.00	4.36	0.71
Q22	Giving reassurance about a clinical procedure	給予病人臨床程序的保證	1.00	4.18	0.85
Q23	Providing privacy to a patient	確保病人的私隱	1.00	4.52	0.65
Q24	Being cheerful with a patient	歡容熱情面對病人	1.00	4.41	0.69
Q25	Observing the effects of a medication on a patient	觀察藥物對病人的影響	1.00	4.37	0.74

**Table 3:** The factor loadings of the CDI in EFA.

Item	Factor Loadings		
	Nurturance	Collaboration	Skill
Q1	0.708	0.196	0.330
Q3	0.702	0.190	0.275
Q4	0.785	0.153	0.282
Q6	0.665	0.213	0.493
Q7	0.709	0.258	0.280
Q8	0.707	0.382	0.205
Q10	0.639	0.464	0.253
Q11	0.756	0.439	0.196
Q13	0.758	0.465	0.178
Q23	0.702	0.428	0.261
Q24	0.567	0.610	0.246
Q12	0.496	0.571	0.264
Q15	0.593	0.543	0.299
Q16	0.157	0.698	0.124
Q17	0.473	0.555	0.436
Q19	0.235	0.685	0.294
Q21	0.552	0.568	0.303
Q22	0.291	0.586	0.474
Q2	0.171	0.085	0.819
Q5	0.600	0.167	0.547
Q9	0.280	0.346	0.729
Q14	0.403	0.454	0.606
Q18	0.331	0.440	0.681
Q20	0.412	0.492	0.555
Q25	0.465	0.428	0.578

Note: KMO = 0.97, Bartlett’s test of spherical significant with a *p*-value of <0.001.

**Table 4:** Cronbach’s alpha for each factor of the CDI.

Factor	Cronbach’s Alpha	Number of Items
Nurturance	0.95	11
Collaboration	0.89	7
Skill	0.92	7
Overall	0.97	25

## Data Availability

All data that support the findings of this study are available from the corresponding author upon reasonable request.
